# Staphylococcus aureus Decolonization Protocol Decreases Surgical Site Infections for Total Joint Replacement

**DOI:** 10.1155/2010/924518

**Published:** 2010-12-20

**Authors:** Scott Hadley, Igor Immerman, Lorraine Hutzler, James Slover, Joseph Bosco

**Affiliations:** NYU-Hospital for Joint Diseases, 301 East 17th Street New York, NY 10003, USA

## Abstract

We investigated the effects of implementation of an institution-wide screening and decolonization protocol on the rates of deep surgical site infections (SSIs) in patients undergoing primary knee and hip arthroplasties. 2058 patients were enrolled in this study: 1644 patients in the treatment group and 414 in the control group. The treatment group attended preoperative admission testing (PAT) clinic where they were screened for MSSA and MRSA colonization. All patients were provided a 5-day course of nasal mupirocin and a single preoperative chlorhexidine shower. Additionally, patients colonized with MRSA received Vancomycin perioperative prophylaxis. The control group did not attend PAT nor receive mupirocin treatment and received either Ancef or Clindamycin for perioperative antibiotic prophylaxis. There were a total of 6 deep infections in the control group (1.45%) and 21 in the treatment group (1.28%); this represented a decrease of 13% (*P* = .809) in the treatment versus control group. This decrease represented a positive trend in favor of staphylococcus screening, decolonization with mupirocin, and perioperative Vancomycin for known MRSA carriers.

## 1. Introduction

Prosthetic joint replacement is a reliable pain-relieving procedure that is in increasing demand with the aging population. Infections associated with prosthetic joints cause a significant morbidity to the patient and a large burden on the health care budget [1–3]. The universal use of preoperative antibiotics and laminar air flow in the operating room has reduced the number of infections associated with primary prosthetic joint replacements to less than 2% [4]. The Centers for Disease Control's National Nosocomial Infections Surveillance (NNIS) system, reports that among surgical patients surgical site infections (SSIs) are the most common nosocomial infection, accounting for 38% of all such hospital acquired infections [5–7]. Furthermore, Staphylococcus aureus is the most commonly identified pathogen in all SSIs at a rate of 25% [5]. Methicillin-resistant Staphylococcus aureus (MRSA) SSIs are an increasing health problem in the United States [8]. According to one study, 57% of nosocomial infections with Staphylcoccus aureus are resistant to methicillin [8]. The ecologic niche of S. aureus is the anterior nares, and at any given time, 25% to 30% of the population is colonized. Patients who are colonized are at a 2 to 9 times higher risk for staphylococcal infections after surgical procedures than are those noncolonized [9]. 

Mupirocin is a topical antibiotic that decolonizes the anterior nares [3, 10]. Intranasal mupirocin prevents postoperative Staphylococus aureus infections in patients that were colonized with S. aureus prior to surgery [3]. In a randomized, double-blinded study of orthopaedic patients the use of mupirocin was found to reduce the rate of endogenous S. aureus infections to 5 times lower than that of the placebo group [11]. Recent literature has demonstrated the cost-effectiveness of universal S. aureus detection in high-risk patient populations [3, 9, 11–13]. 

We implemented a screening, decolonization, and antibiotic prophylaxis protocol for all patients that have elective surgery at our institution. The purpose of this study is to compare the effect of our MRSA decolonization protocol on the rates of SSIs in patients who underwent primary knee and hip arthroplasties from 2007 to 2009 versus those patients who did not follow this protocol.

## 2. Material and Methods

### 2.1. Selection Criteria

All patients undergoing primary total knee or total hip arthroplasty from November 2007 through June 2009 at NYU Hospital for Joint Diseases were enrolled in the study. Each patient was included once even if bilateral surgeries were performed. Patients undergoing a revision operation or nonprosthetic hip or knee surgery were excluded from the study. All eligible patients that attended preadmission testing clinic (PAT) at NYU-HJD were considered part of the treatment arm. The remaining eligible patients that did not attend preadmission testing at NYU-HJD made up the control group. Both groups underwent total joint surgery during the same time period. The IRB committee at NYU School of Medicine approved the study protocol.

### 2.2. Treatment

**Figure 1:**
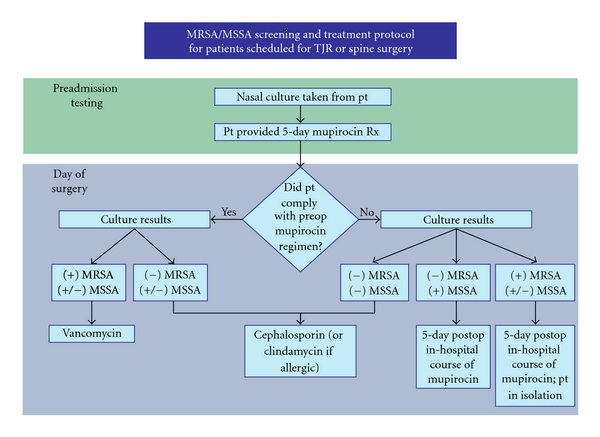
MRSA/MSSA screening and treatment protocol.

Patients in the treatment arm attended PAT clinic within 30 days of their surgery. During the visit, swabs of both nares were obtained and sent off to NYU microbiology laboratory for routine cultures for the presence of staph species. Irrespective of the culture result all patients that went through PAT received a prescription for a 5-day course of 2% Mupirocin nasal ointment and a single preoperative chlorhexidine shower [5, 8, 9]. Nasal screening results were available for all treated patients on the day of surgery, and MRSA positive carriers were given perioperative Vancomycin 1 gram every 12 hours starting at least 30 minutes before incision and lasting for 24 hours. The MRSA/MSSA status of the non-PAT control group was unknown and, therefore, either Ancef or Clindamycin prophylaxis was administered. The surgical technique, implants and postoperative care were similar in both groups. (See Figure 1).

### 2.3. Outcome

The primary outcome was the rate of SSI. Secondary outcomes were the prevalence of MSSA/MRSA in the study population, rate of SSIs due to S. aureus, and the rate of endogenous S. aureus SSIs. All patients were followed for one year for postoperative infection. SSIs were classified using the Centers for Disease Control criteria [7]. Only deep incisional SSIs were considered clinically relevant and considered in the analysis.

### 2.4. Statistical Analysis

Statistical analysis was performed using Fisher's exact test.

## 3. Results

During the study period, 2058 patients were included in the study. There was complete followup in this retrospective study of prospectively collected data. The total number of treatment patients was 1644 patients (80%) and 414 (20%) in the control group. At the onset of the study the proportion of the PAT treatment group to the control group was roughly equal. However, as the study progressed the protocol was quickly adopted by all surgeons and the non-PAT patients decreased significantly.

Of the 1644 patients screened, 58 (3.5%) had positive nasal swabs for MRSA. MSSA was positive in 351 patients. (21.4%) At the surgical visit, the OR nurse asked about treatment compliance, and there was a self-reported 96.4% compliance rate with mupirocin nasal treatment and a 98.8% compliance rate with chlorhexidine shower. 

There were a total of 6 deep infections in the control group (1.45%) and 21 in the treatment group (1.28%). All but one of the 27 infections had a positive culture for at least one microorganism. There was 1 MRSA infection in the control group (0.24%) and 3 MRSA infections in the treatment group (0.30%); 1 patient was found to be a carrier of MRSA on initial PAT and later developed a MRSA infection postoperatively. Those microorganisms isolated in addition to Staphylococcus species included Streptococcus species,Pseudomonas, and E. coli. (See Table 1).

## 4. Discussion

There is evidence that staphylococci colonization is a risk factor for surgical site infection. Studies have shown that 10%–15% of healthy adults carry Staphylococcus aureus in their nares; this figure rises to 20%–35% in hospital personnel [9]. It has been noted that a high-level of nasal carriage of Staphylococcus aureus is the most important and only significant risk factor of developing a surgical site infection [14]. A randomized double-blinded, placebo-controlled multicenter trial showed a decrease in the rate of deep surgical site infection of 4.3% to 3.4% from 7.7% with decolonization of nasal carriers of MSSA [12]. 

In our patient population, staphylococci decolonization led to a 13% decrease (*P* = .809) in deep surgical site infections. These findings did not reach statistical significance, but they represented a positive trend towards the efficacy of a decolonization program in decreasing infections. The primary weakness of this study was the small non-PAT control group. When the decolonization protocol was first introduced there were a significant number of patients who attended outside clinics for preoperative clearance. However this control group quickly diminished as surgeons at our institution realized the utility of PAT clinic. This fact combined with the already low infection rate in primary total joint replacement surgery made it impossible to reach statistical significance from one center. We performed a power analysis and determined that a sample size of 57,604 patients in each group would be required for statistical significance given the low rate of infections in the control group. Another limitation of this study is that the individual components of the decolonization protocol (i.e., mupirocin decolonization, chlorhexidine shower, and prophylactic antibiotics) were not individually tested. We hypothesize that there is a synergistic benefit to each of these steps, but further studies would be needed to determine their individual effect on the primary outcome.

Despite its limitations, to our knowledge, this is the largest study on the effect of a decolonization protocol with mupirocin in an elective orthopaedic population. A similar study in the Orthopaedic trauma literature identified risk factors associated with MRSA infections but had a baseline infection rate that was doubled that of typical total joint surgery [15]. One should use caution when applying the results of the trauma literature to elective primary joint replacement surgery [1, 15, 16]. To date the evidence for the effectiveness of staphylococcus decolonization protocols to decrease infection in total joint patients has been split. Several level III studies conclude that there is a benefit to staphylococcus decolonization in elective orthopaedic surgery, while one level I study refutes it [13, 17, 18]. One study looked at 1495 consecutive patients who underwent total joint replacement in a 2 years period compared to a historical control and concluded that the nasal decolonization for S. aureus resulted in a fourfold decrease in S. aureus surgical site infections for patients colonized with S. aureus [18]. The only randomized, double-blinded, and placebo-controlled study of the use of mupirocin in an elective orthopaedic population yielded mixed results [11]. They found a fivefold lower rate of SSIs caused by S. aureus in the mupirocin group compared to placebo; however, there was also a much higher rate of deep SSIs in the treatment group compared to placebo [11]. It should be noted that all were single-center studies and did not have large enough sample sizes to yield statistical significance.

## 5. Conclusion

The role of mupirocin in the elective orthopaedic population has not been well studied. At this time, there is no evidence that decolonization will reduce the deep infection rate or overall surgical site infection rate. Although this is the largest retrospective review of the effect of MRSA decolonization on infection in total joint surgery, it is underpowered to reach any significant findings. Due to the low rate of infections in primary total knee and hip surgeries, it is impossible for any single institution to enroll a sufficient number of patients for trends to reach statistical significance [4, 19, 20]. Two large double-blinded, randomized studies of general surgery patients compared analogous mupirocin decolonization protocols to placebo and concluded that there is a significant decrease in the rate of nosocomial infections among high-risk patients with nasal carriage of S. aureus [3, 12]. Additionally, there was a clear costeffective benefit for decolonization of those patients colonized with MRSA [12]. Our group recently published findings on the costeffectiveness of a Staphylcoccus screening and decolonization protocol in the high-risk Orthopaedic patients [21]. We reported that only a modest reduction in the surgical site infection rate was necessary to be cost savings to the hospital. The trend we observed in this study although not statistically significant would clearly be financially significant [21]. It is the authors' opinion that with a sufficiently large elective orthopaedic population, similar results and conclusions would be reached. Our findings in this study add to a growing body of evidence that the decolonization of S. aureus decreased surgical infections. Clinicians may want to consider preoperative decolonization given the risk and significant consequences of infection.

## Figures and Tables

**Table 1:** 

	*# *Patients	Deep SSIs	Percentage	MRSA infections	Percentage
Control	414	6	1.45%	1	16.6%
Treatment	1644	21	1.28%	3	14.3%

Totals	2058	27	1.31%	4	14.8%
